# A Longitudinal Study of the Influence of the COVID-19 Pandemic on Anxiety and Stress among Medical University Students

**DOI:** 10.3390/jcm13030890

**Published:** 2024-02-03

**Authors:** Oskar Wróblewski, Kaja Michalczyk, Mateusz Kozłowski, Katarzyna Nowak, Anita Chudecka-Głaz, Edyta Skwirczyńska

**Affiliations:** 1 Collegium Humanum, 03-772 Warsaw, Poland; oskraw@gmail.com; 2 Department of Gynecological Surgery and Gynecological Oncology of Adults and Adolescents, Pomeranian Medical University in Szczecin, 70-111 Szczecin, Poland; anitagl@poczta.onet.pl; 3 Department of Reconstructive Surgery and Gynecological Oncology, Pomeranian Medical University, 70-204 Szczecin, Poland; mtkoozo@gmail.com (M.K.); kn13222@gmail.com (K.N.); 4 Department of History of Medicine and Medical Ethics, Pomeranian Medical University in Szczecin, 70-204 Szczecin, Poland; socjoterapia@onet.pl

**Keywords:** COVID-19, pandemic, anxiety, stress, STAI, PSS-10, mental health

## Abstract

(1) **Background:** The COVID-19 pandemic was declared an international health emergency by the World Health Organization. The dramatic, widespread transmission of the virus, high mortality, and lack of specific drugs caused the development of panic attacks and anxiety. Healthcare professionals, including medical students, were challenged to provide medical care to patients in need, often exposing themselves to the virus. (2) **Methods:** This study aimed to assess anxiety and stress levels in medical students, both at the beginning and after the end of the COVID-19 pandemic. The STAI and PSS-10 questionnaires were used. (3) **Results:** This study showed a decrease in anxiety levels among medical students after the end of the COVID-19 pandemic. In both 2020 and 2022, female students were found to experience higher stress levels than male students. Anxiety levels were similar among students of different medical-related faculties. (4) **Conclusions:** The COVID-19 pandemic caused stress and anxiety among medical students, which decreased throughout the duration of the pandemic.

## 1. Introduction

In January 2020, the WHO (World Health Organization) declared the COVID-19 pandemic an international health emergency. According to the WHO, since the beginning of the pandemic, there have been over 11,300,000 confirmed cases of COVID-19 infection, of which over 530,000 people have died [1,2]. The first cases of the disease occurred in China in late 2019, while the first cases of COVID-19 in Poland occurred in March 2020 [3,4]. Following the example of other countries around the world, the Polish government introduced numerous restrictions for citizens to spread the burden of infections over an extended time, thus giving healthcare facilities time to prepare for the increase in the number of patients. The restrictions introduced by the government concerned various spheres of life, affecting economic and social development; in addition to the mandatory wearing of masks, an isolation order was imposed on citizens, the main element of which was the closure of public places, movement only to work, hourly restrictions on shopping, maintaining distance between people, and a ban on movement [5]. Even though the pandemic continued to rage, it posed an enormous risk, especially to elderly patients with comorbidities. The COVID-19 pandemic posed severe problems to healthcare professionals and medical students, resulting not only in medical issues in general health but also highly affecting mental health. The robust, widespread transmission of the virus, high mortality, and lack of specific drugs or vaccines resulted in the development of panic attacks and anxiety.

Medical students were a particular group of students whose lives were especially affected by the COVID-19 pandemic. Together with healthcare professionals, they were working on the frontline of the COVID-19 pandemic, often prioritizing others before themselves and forgetting about their own needs. They had to face extreme situations and emotions, working over the limits to help the fight against the pandemic. Medical students are a particular group who, even under normal, non-pandemic conditions, are at higher risk of developing anxiety disorders than the general population. University carries a considerable amount of demands, resulting in significant psychological stress, which may affect one’s academic performance, physical well-being, and mental health. The study plans are always filled with classes, starting from clinical rotations or laboratory classes in the early morning and finishing with university lectures in the late evening. Until the beginning of the pandemic, all of these classes were stationary. During clinical classes and seminars, the students were subdivided into subgroups of approximately 20 people. At the same time, all of the lectures were held in big university classrooms, hosting approximately 200–300 students at a time. The COVID-19 pandemic greatly affected the study mode. Students had to face the postponed start of classes in the spring semester of 2020 and become accustomed to online classes like other students. Planned internships and clinical classes were initially ceased to limit patient contact and potential COVID-19 infection spread. However, many students became actively involved in measures against the COVID-19 pandemic, helping hospitals with patient management and stratification, COVID-19 testing, and COVID-19 vaccinations, and providing patient care in COVID-19 departments. 

The characteristics of the COVID-19 pandemic and the fear it caused to the general population caused public anxiety, panic attacks, and increased depression rates. Moreover, limited information to the general population on the management of the COVID-19 pandemic and its spread, as well as the lack of psychological relief measures to cope with the pandemic, aggravated the negative outlook of the pandemic and the negative emotions experienced by the general public. 

The pandemic affected different aspects of everyday life, including the following:

- A change in health-related behaviors through increased safety measures, orders to wear masks and the inconvenience caused by it, and the need for increased hand disinfection;
- Difficulties in accessing primary and specialistic medical care;
- The introduction of various quarantine policies, limiting peoples’ mobility and freedom of travel within and outside of the country;
- Disinformation and limited information to the general population regarding the spread of the virus and related mortalities;
- The economy aspect, limited store opening hours, changes in employment and income, and limits on general needs, food, and essential hygienic equipment.

The restrictions introduced and the stress related to the pandemic situation may have affected the population’s mental health [6]. There is a limited number of publications relating to the mental health of specific social groups. Due to the unprecedented scale and uniqueness of this phenomenon around the world, for the first time in modern history, people experienced so many restrictions, causing a sense of threat and resulting in the need to activate strategies to cope with difficult situations and internal and external resources. In the social dimension, everyone became a threat to everyone, regardless of religion, social status, or place of work. 

This longitudinal study was created to understand the psychological performance of college students during the different phases of the COVID-19 pandemic. This study assessed anxiety and stress among medical university students at two different time points: at the beginning of the COVID-19 pandemic and after two years, when most of the government-based pandemic measures were over. The primary objective of this study was to confirm an increased incidence of depression and anxiety symptoms among university students during the COVID-19 pandemic. The secondary objective was to assess the long-term influence of the pandemic on the anxiety and depression symptoms of university students once the pandemic ended. In our study, we used the anxiety measurement tool. It is a questionnaire used for both individual and group testing. It is a suitable screening test to detect people with both very high and very low levels of anxiety. The STAI tool consists of two scales, each containing 20 questions to measure state anxiety and trait anxiety [7].

## 2. Materials and Methods

This study examined anxiety and stress levels among students of the Pomeranian Medical University, Szczecin, Poland. This research was conducted in accordance with the Declaration of Helsinki and approved by the Ethics Committee of the Pomeranian Medical University in Szczecin (protocol code KB-0012/97/2020). The study population included students from the Faculties of Health Sciences, Pharmacy, and Medicine and Dentistry). This was a prospective, observational, longitudinal study, conducted at two points in time: the first in 2020, at the beginning of the COVID-19 pandemic, and later, in 2022, after the end of all of the restrictions. The study participants were selected from defined populations of students with similar exposures over time. The majority of the study participants were questioned at both timepoints; however, some might have differed over time. Not all of the participants returned the questionnaires in time. The State-Trait Anxiety Inventory (STAI) and Perceived Stress Scale (PSS-10) questionnaires were used to determine stress and anxiety. In 2020, 621 students participated in the study; in 2022, 489 questionnaires were completed. An online questionnaire was distributed through the university e-mail platform to all grades of Pomeranian University students. The survey was distributed in the Polish language. The study inclusion criteria were >18 years of age, being an active student of the Faculty of Health Sciences, Faculty of Pharmacy, or Faculty of Medicine and Dentistry of the Pomeranian Medical University in Szczecin. Only fully completed questionnaires were evaluated. The questionnaires were voluntary and anonymous. 

The participants were asked to complete online questionnaires regarding socio-demographic data, perceived stress, and anxiety levels, with stress understood as a temporary and situationally conditioned state of the individual and anxiety understood as a relatively permanent personality trait. In addition to the STAI anxiety measurement tool, this study used a structured questionnaire containing questions about the students’ demographics, including their gender, place of residence, subjective assessment of one’s health, nature of employment, and financial resources, as well as their behaviors and personal opinion regarding the restrictions introduced by the government. The students were also asked if they complied with the governments’ restrictions and whether they would like to attend stationary university classes during the pandemic. 

The respondents were asked to complete an online survey made available two weeks after the government introduced restrictions due to the coronavirus pandemic. This research was conducted as an electronic survey posted on the Google Forms platform. The subjects were informed about the lack of a time limit and the possibility of withdrawing from the study at any time. After being informed about the purpose of the study, all participants voluntarily provided their informed consent to participate in the study. 

The questionnaire was designed to last a maximum of about 20 min. The first part of the survey consisted of personal details and questions about the pandemic situation in the country. In the second part, the subjects completed a questionnaire consisting of 40 statements divided into two subscales of 20 questions, each used to measure anxiety as a state and anxiety as a trait. The first 20 statements were related to anxiety as a state (s-STAI), which assesses a transient emotional state; the second 20 statements were related to the anxiety-as-trait subscale (t-STAI), which, in general, considers a relatively stable propensity towards anxiety. The questionnaire used a 4-point Likert scale (0—almost never/not at all; 1—sometimes/somewhat; 2—often/moderately so; 3—almost always/very much so). 

This study also included the PSS-10 (Perceived Stress Scale) questionnaire. A version from 2009, adapted to the Polish language, was used. The PSS-10 is used to measure perceived stress. It contains ten questions on various subjective feelings related to personal problems and events, behaviors, and ways of coping. For each question, one of the following alternatives can be selected: 0—never; 1—almost never; 2—sometimes; 3—fairly often; 4—very often. The PSS-10 scores range from 0 to 40, with higher scores indicating higher perceived stress. 

Statistical analyses were conducted using Statistica 10.0. The following descriptive statistics were used to present the variables: the arithmetic mean, number of valid cases, minimum, maximum, median, dominant, and standard deviation. A structure index including percentages was also used, as well as mathematical statistics, including tests of significance of differences, non-parametric correlations, and tests of distribution fit. A normal distribution was not confirmed in the study groups, necessitating the use of the non-parametric Shapiro–Wilk test. Statistical inference was based on Student’s *t*-test analysis of variance for independent groups. The presence and significance of correlations were tested, starting with Chi-square independence tables, and then assessed using the Spearman correlation coefficient. A statistical significance level of *p* < 0.05 was adopted. 

## 3. Results

### 3.1. Socio-Demographic Data

In this study, the male population accounted for 32.7% of the students assessed in 2020 and 38.2% of the students questioned in 2022. The female population equaled 67.3% of students in 2020 and 63.8% in 2022. The majority of students participating in the study attended the Faculty of Medicine and Dentistry (58.9% in 2020, 54.8% in 2022). The Faculty of Health Sciences was represented by 30.3% of students in 2020 and 32.3% in 2022, while the Faculty of Pharmacy by 10.8% in 2020 and 12.9% in 2022. The population of students not only varied based on the faculty but also based on the year of study. In 2020, the highest percentage was represented by the students attending the sixth year of their studies, while in 2022, the majority was represented by fifth-year students. The detailed results are presented in the table below (Table 1).

In 2020, 81% of students participating in the study admitted to adhering to the recommended restrictions. The majority of students (63.1%) wanted to participate in stationary university classes. As many as 73% of participants admitted to experiencing some problems with concentration. Also, 62% of students were noted to have poorer grades. In the follow-up study conducted in 2022, 85.9% of participants complied with the recommended government and university restrictions. Almost all students (93.7%) declared their willingness to participate in stationary classes rather than online. The majority of students (86.7%) admitted to experiencing problems with concentration. 

### 3.2. The STAI Questionnaire

The STAI questionnaire was used to assess the students’ anxiety levels at the beginning and after the end of the COVID-19 pandemic. In 2020, the mean total anxiety level was 44.94 ± 14.05, while in 2022, it lowered to 43.06 ± 12.63. Also, other decreasing trends were found when evaluating the STAI questionnaire subscales. The STAI score for anxiety as a trait in 2020 equaled 43.18 ± 12.89; in 2022, it lowered to 41.74 ± 11.81. A similar observation was made for the STAI score for anxiety as a state, with 46.11 ± 11.56 in 2020 and 43.98 ± 12.25 in 2022. The results of specific STAI questionnaires are demonstrated in Table 2.

The STAI scores were analyzed based on socio-demographic data. There were no statistically significant differences between the male and female student populations in 2020 and 2022. At the beginning of the COVID-19 pandemic, students in their early university training years (years I–III) experienced significantly higher anxiety than students in later training (years IV–VI); *p* = 0.014. There were no differences in STAI anxiety scores after the end of the COVID-19 pandemic between the students in years I–III and IV–VI. 

During the two time points of this study, there were significant differences between the anxiety levels experienced by medical and dentistry students when compared to the students attending the faculty of pharmacy, with pharmacy students experiencing higher anxiety (*p* = 0.003 and *p* = 0.043, accordingly). The lowest anxiety STAI scores were experienced by medical and dentistry students, both at the beginning and at the end of the COVID-19 pandemic. 

Generally, all of the assessed population subgroups experienced lower anxiety after the COVID-19 pandemic than in its beginning. The specific results are listed in Table 3. 

### 3.3. The PSS-10 Score

As a part of this study, we also conducted an analysis on the influence of socio-demographic characteristics on the PSS-10 score. A general decrease in PSS-10 scores was observed between 2020 and 2022 in the specific subgroups. There were no statistically significant differences in PSS-10 scores between female and male students at the beginning or at the end of the COVID-19 pandemic. In 2020, a significant difference in PSS-10 scores was observed based on the year of study. Students in early university years experienced higher PSS-10 scores than students in years IV–VI of university. However, there were no significant differences in PSS-10 scores after the pandemic. The only statistically significant PSS-10 score difference based on the faculty was found between pharmacy and health sciences students, with pharmacy students experiencing higher stress levels on the PSS-10 scale. Students living in dormitories experienced the lowest stress at the beginning and end of the COVID-19 pandemic. The specific results are listed in Table 4.

### 3.4. Anxiety and Financial Resources

An analysis using Student’s *t*-test for independent samples showed that the average state anxiety of students with fewer financial resources (with financial resources for less than five weeks) was statistically significantly higher (mean = 49.40; SD 12.13) than the average anxiety of people in possession of greater financial resources (mean = 45.02; SD = 12.87); *p* < 0.001. In the case of trait anxiety, the analysis showed that the mean anxiety trait of people with fewer financial resources is statistically significantly higher (mean = 48.25; SD = 10.39) than in people with greater financial resources (mean = 44.50; SD = 10.67), *p* < 0.001.

We also performed separate analyses for the male and female populations of students. Women with fewer financial resources experienced statistically significantly higher state anxiety (mean = 50.60; SD = 11.83) than women with greater financial resources (mean = 46.82; SD = 12.89), *p* < 0.001. Similar findings were found for the male student population (mean = 44.72; SD = 11.77, mean = 39.88; SD = 11.37), accordingly; *p* < 0.001. 

## 4. Discussion

The COVID-19 pandemic has affected many areas of human life, with physical and mental health at the top of the pyramid. The economy, finances, and interpersonal relationships were also affected [8,9,10,11]. In the beginning, as they were not life-threatening, these effects were a minor issue; however, with the prolonged duration of the COVID-19 pandemic, they became more and more bothersome. The uncertainty related to the pandemic and its duration, as well as the dramatic changes to peoples’ behavior—the fear of others and the need for social distancing—have profoundly impacted people’s mental health [12,13,14]. 

University itself carries a considerable amount of demands resulting in significant psychological stress, which may affect one’s academic performance, physical well-being, and mental health. Our results confirmed the hypothesis that the COVID-19 pandemic increased the anxiety and stress levels during the among the students of the Pomeranian Medical University. These stress levels were especially raised at the beginning of the pandemic and declined with its duration.

A systematic review by Dyrbye et al. [15] reported a higher prevalence of anxiety symptoms among the medical student population compared to non-medical students. During the COVID-19 pandemic, medical students were asked to provide additional help to medical professionals in providing COVID-19 protective measures, in the triage of patients upon hospital admission, or by working at special departments created for COVID-19 patients. They also had to temporarily cease university classes, transition to online learning schemes, and enact COVID-19-induced lifestyle changes. Even though medical students may seem to be at the highest risk of developing stress and anxiety, the results of our study demonstrate lower stress and anxiety levels both at the beginning and at the end of the COVID-19 pandemic among medical and dentistry students compared to the students of other faculties. This group of students might have been less prone to stress and anxiety disorders due to closer hands-on contact with medical institutions and active participation in the fight against pandemic, allowing them to believe that the pandemic would finally end. This group of students was indeed more prepared for a pandemic due to a greater knowledge of viral diseases, their spread, and potential pandemic management, as well as an awareness of the SARS-CoV-2 [16].

In our study, in order to assess students’ perceived stress levels, we used the Polish adaptation of the STAI questionnaire. Among the assessed students, we did not find any differences in general stress levels between the male and female populations of students. Our results are similar with the ones obtained by Yildirim et al. [17], who conducted a study on Turkish dentistry students. Also, Lie et al. found no differences between male or female students. Students’ majors did not influence the rates of state anxiety and trait anxiety [18]. In our study, we tried to evaluate if the year of the study (preclinical vs. clinical students) influenced the perceived level of stress experienced during the COVID-19 pandemic. Preclinical students (year I–III) were found to have both higher STAI and PSS-10 scores. Also, other authors have investigated for STAI score differences between preclinical and clinical students; however, they did not find any differences [17]. 

A study by Loda et al. was conducted on a population of medical students in Germany. The study showed the mean STAI sum score to significantly correlate with students’ academic lives, and not their personal ones. The main issues regarding classes during the COVID-19 pandemic considered a lack of adequate protection during medical classes in hospital units (masks, gloves, glasses, protective suits) and a lack of access to study materials. The students also had to deal with the disadvantages of online learning, such as a lack of contact with patients and, thus, training in clinical skills, or a lack of contact with other students [19]. In our survey conducted in 2020, at the beginning of the COVID-19 pandemic, 63.1% of participants wanted to participate in stationary activities, while in 2022, as many as 93.7% of students wanted to return to stationary university classes in hospitals. Students also lacked contact with their peers and expressed a willingness to have direct contact with other students. It should be noted that, as one of the few medical universities in the country, after several months of suspension of full-time classes, Pomeranian Medical University returned to stationary classes and continued them throughout the pandemic. Excessive online training and a lack of direct patient contact could be a potential problem and contribute to limited skills and further difficulties in patient contact, especially among students who are about to finish their medical school training. Also, a complete lockdown and lack of face-to-face intrapersonal contact could contribute to the development of mental health issues. Borisenko et al. observed similar findings as they described the change from in-person to online teaching to significantly impact students’ mental health. Students were found to experience increased anxiety and depression [20]. Studies conducted during the COVID-19 pandemic showed young people to be especially vulnerable to increased psychological stress, probably due to a stronger need for social interactions, with young women seeming to be more vulnerable than young men to mental health problems [21].

Aslan et al. conducted a survey-based study on a population of Turkish students; 70% of respondents reported high stress (PSS-10 scores: 20–40 points) in the context of the COVID-19 pandemic, while only a little over 5% reported low stress (PSS-10: 0–13 points). In terms of gender, higher PSS-10 scores, and thus higher stress levels, were reported by female students. Also, more than half of the students presented clinical symptoms of generalized anxiety disorder (GAD) during the survey, and 60% of the students presented with depressive symptoms [22].

A different study conducted by Zhan et al. described stress and anxiety levels in a medical and non-medical cohort of Chinese students. Female students reported higher stress and anxiety (assessed by the PSS-10 questionnaire). The study showed lower stress and anxiety than non-medical students, demonstrating that medical students may be more prone to stress than other students. No differences in stress levels were shown between the final-year students compared to the students earlier in their careers [23]. 

Our study was conducted at two time points: in 2020 (at the beginning of the COVID-19 pandemic) and 2022 (after the pandemic). There are limited studies assessing anxiety and stress levels throughout the COVID-19 pandemic. A study by García-González et al. was performed to evaluate anxiety levels in nursing students. It was also conducted at two time points, yet over a shorter period of time, as the surveys were conducted only four weeks apart [24]. A statistically significant increase in anxiety levels in students in week four of the study was observed. Female students were found to experience higher levels of anxiety than men in both timeframes. However, the increase in anxiety in men was higher [24]. The secondary objective of the study was to assess the long-term influence of the pandemic and the depression symptoms of university students after the end of the pandemic. In our research, we found significantly lower anxiety and stress levels in students after the end of COVID-19 and COVID-19-associated restrictions when compared to 2020, when the COVID-19 pandemic had just started. It might have been interesting to conduct the study survey at more time points to evaluate the changes in stress levels during the entire duration of the COVID-19 pandemic. In our study, both in 2020 and 2022, female students experienced higher stress levels than male students. The decrease in stress levels was higher among female students than in males. Also, similar to the Chinese study, the stress levels in preclinical students were similar to those of clinical students at both study timepoints.

Considering the place of residence, students who lived at home had a lower mean STAI score than students who lived in rental apartments, i.e., students who might have changed their place of residence for university purposes and came from different cities. As traveling became an issue during the COVID-19 pandemic, students might have had additional anxiety regarding the lives of their families, as they were unable to visit them as often and travel to them as before. Similar results were obtained in a study on Chinese college students studying in China or the USA. Those who did not leave the country were found to have lower STAI scores than those who traveled abroad [25]. 

In our study, we also tried to assess whether the level of financial security translates to the level of anxiety. The obtained results indicate that people who have financial security for a longer period of time experience significantly lower levels of anxiety, both as a state and a trait. Cross-gender research has shown that men with financial security for more than five weeks experience lower levels of anxiety than women with security for the same period. In our study, women tended to experience higher levels of anxiety with regard to their financial situation more than men. The COVID-19 pandemic exerted a significant impact on our lives, not only physically, but also impacting different aspects of our lives. Initially, the financial aspect seemed of low importance; however, with the increasing lockdown, not all of the financial sectors were working as before. Multiple people lost their jobs or had to rebrand to maintain their salaries. Research by Tull et al. demonstrated that forced isolation during the COVID-19 pandemic caused higher levels of anxiety and greater concerns regarding one’s financial situation [26].

Multiple studies have highlighted that personal experiences related to COVID-19, COVID-19-associated restrictions, lifestyle modifications, COVID-19 infections, and mortality in acquaintances have an elevated risk of emotional disorder symptomatology and increased rates of stress and anxiety among all populations [18,27]. The results of our study show increased stress and anxiety levels among all students despite their faculty. The COVID-19 pandemic altered students’ everyday functioning, resulting in psychological changes and causing an increased demand for mental health services. Psychological support or interventions should be offered to university students. 

## 5. Conclusions

Our study demonstrates increased anxiety and stress levels among university students during the COVID-19 pandemic. However, they decreased significantly in the post-pandemic world compared to the beginning of the COVID-19 pandemic. A greater decrease in anxiety and stress levels was noticed among female students. Anxiety levels were similar regardless of the studied faculty. The COVID-19 pandemic significantly affected students’ mental health. Changes to their everyday lifestyle, university schedules, and study mode affected both stress levels and anxiety among all of the students, despite their faculty. Psychological help should be offered to cope with life alternations that cause increased stress and anxiety.

## Figures and Tables

**Table 1:** The characteristics of study population.

	Characteristic	N of Students in 2020 (*n* = 621)	N of Students in 2022 (*n* = 489)
Sex	Male	203 (32.7%)	177 (36.2%)
	Female	418 (67.3%)	312 (63.8%)
Faculty	Health Sciences	188 (30.3%)	158 (32.3%)
	Pharmacy	67 (10.8%)	63 (12.9%)
	Medicine and Dentistry	366 (58.9%)	268 (54.8%)
Year of study	I	58 (9.3%)	77 (15.8%)
	II	49 (7.9%)	83 (17.0%)
	III	101 (16.3%)	88 (18.0%)
	IV	98 (15.8%)	68 (13.9%)
	V	141 (22.7%)	94 (19.2%)
	VI	174 (28%)	79 (16.2%)
Place of residence	Dormitory	359 (57.8%)	291 (59.5%)
	Rental	187 (30.1%)	129 (26.4%)
	Family home	75 (12.1%)	69 (14.1%)

**Table 2:** Mean STAI questionnaire scores.

	2020		2022	
	Mean ± SD	*p*-Value	Mean ± SD	*p*-Value
STAI (total score)	44.94 ±14.05	0.067	43.06 ± 12.63	0.003
STAI (anxiety as a trait)	43.18 ± 12.89	0.023	41.74 ± 11.81	0.003
STAI (anxiety as a state)	46.11 ± 11.56	0.058	43.98 ± 12.25	0.118

SD—standard deviation.

**Table 3:** The STAI questionnaire based on socio-demographic characteristics.

Characteristic		STAI Score in 2020		STAI Score in 2022	
		Mean ± SD	*p*-Value	Mean ± SD	*p*-Value
Sex	Female	46.34 ± 13.25	0.059	42.06 ± 10.92	0.142
	Male	42.80 ± 12.89		42.12 ± 11.05	
Year of study	I–III	43.18 ± 12.47	0.014	41.39 ± 11.01	0.052
	IV–VI	41.25 ± 11.98		40.61 ± 9.90	
Faculty	Medicine and Dentistry vs. Pharmacy	41.58 ± 11.65 vs. 45.90 ± 13.04	0.003	40.19 ± 9.25 vs. 41.75 ± 9.38	0.043
	Medicine and Dentistry vs. Health Sciences	41.58 ± 11.65 vs. 44.15 ± 12.92	0.159	40.19 ± 9.25 vs. 41.17 ± 9.65	0.049
	Pharmacy vs. Health Sciences	45.90 ± 13.04 vs. 44.15 ± 12.92	0.232	41.75 ± 9.38 vs. 41.17 ± 9.65	0.121
Place of residence	Dormitory vs. Rental	40.91 ± 9.84 vs. 44.65 ± 12.15	0.004	39.99 ± 9.03 vs. 41.20 ± 10.12	0.055
	Dormitory vs. Family home	40.91 ± 9.84 vs. 43.17 ± 11.65	0.019	39.99 ± 9.03 vs. 40.76 ± 9.86	0.062
	Rental vs. Family home	44.65 ± 12.15 vs. 43.17 ± 11.65	0.126	41.20 ± 10.12 vs. 40.76 ± 9.86	0.024

**Table 4:** PSS-10 questionnaire results based on socio-demographic characteristics.

Characteristic		PSS-10 Score in 2020		PSS-10 Score in 2022	
		Mean ± SD	*p*-Value	Mean ± SD	*p*-Value
Sex	Female	16.25 ± 4.02	0.056	12.18 ± 3.08	0.101
	Male	12.89 ± 3.15		11.03 ± 2.75	
Year of study	I–III	12.53 ± 2.93	0.045	12.01 ± 2.56	0.057
	IV–VI	11.12 ± 2.04		11.26 ± 2.11	
Faculty	Medicine and Dentistry vs. Pharmacy	13.23 ± 3.67 vs. 16.89 ± 4.11	0.121	10.85 ± 2.83 vs. 11.92 ± 2.95	0.232
	Medicine and Dentistry vs. Health Sciences	13.23 ± 3.67 vs. 12.11 ± 3.45	0.275	10.85 ± 2.83 vs. 11.14 ± 3.03	0.143
	Pharmacy vs. Health Sciences	16.89 ± 4.11 vs. 12.11 ± 3.45	0.047	11.92 ± 2.95 vs. 11.14 ± 3.03	0.328
Place of residence	Dormitory vs. Rental	11.99 ± 2.98 vs. 15.28 ± 3.87	0.021	10.21 ± 2.44 vs. 12.48 ± 3.89	0.033
	Dormitory vs. Family home	11.99 ± 2.98 vs. 13.04 ± 3.11	0.168	10.21 ± 2.44 vs. 11.32 ± 3.21	0.002
	Rental vs. Family home	15.28 ± 3.87 vs. 13.04 ± 3.11	0.049	12.48 ± 3.89 vs. 11.32 ± 3.21	0.043

## Data Availability

All data were collected from the Department of the History of Medicine and Medical Ethics, Pomeranian Medical University, Szczecin. Data are available on request.
